# OutSingle: a novel method of detecting and injecting outliers in RNA-Seq count data using the optimal hard threshold for singular values

**DOI:** 10.1093/bioinformatics/btad142

**Published:** 2023-03-22

**Authors:** Edin Salkovic, Mohammad Amin Sadeghi, Abdelkader Baggag, Ahmed Gamal Rashed Salem, Halima Bensmail

**Affiliations:** College of Science Engineering, Hamad Bin Khalifa University, Doha, Qatar; Qatar Computing Research Institute, Hamad Bin Khalifa University, Doha, Qatar; Qatar Computing Research Institute, Hamad Bin Khalifa University, Doha, Qatar; Department of Computer Sciences, College of Engineering, Qatar University, P.O. Box: 2713, Doha, Qatar; Qatar Computing Research Institute, Hamad Bin Khalifa University, Doha, Qatar

## Abstract

**Motivation:**

Finding outliers in RNA-sequencing (RNA-Seq) gene expression (GE) can help in identifying genes that are aberrant and cause Mendelian disorders. Recently developed models for this task rely on modeling RNA-Seq GE data using the negative binomial distribution (NBD). However, some of those models either rely on procedures for inferring NBD’s parameters in a nonbiased way that are computationally demanding and thus make confounder control challenging, while others rely on less computationally demanding but biased procedures and convoluted confounder control approaches that hinder interpretability.

**Results:**

In this article, we present OutSingle (Outlier detection using Singular Value Decomposition), an almost instantaneous way of detecting outliers in RNA-Seq GE data. It uses a simple log-normal approach for count modeling. For confounder control, it uses the recently discovered optimal hard threshold (OHT) method for noise detection, which itself is based on singular value decomposition (SVD). Due to its SVD/OHT utilization, OutSingle’s model is straightforward to understand and interpret. We then show that our novel method, when used on RNA-Seq GE data with real biological outliers masked by confounders, outcompetes the previous state-of-the-art model based on an *ad hoc* denoising autoencoder. Additionally, OutSingle can be used to inject artificial outliers masked by confounders, which is difficult to achieve with previous approaches. We describe a way of using OutSingle for outlier injection and proceed to show how OutSingle outperforms its competition on 16 out of 18 datasets that were generated from three real datasets using OutSingle’s injection procedure with different outlier types and magnitudes. Our methods are applicable to other types of similar problems involving finding outliers in matrices under the presence of confounders.

**Availability and implementation:**

The code for OutSingle is available at https://github.com/esalkovic/outsingle.

## 1 Introduction

Recent research efforts have made evident that whole-genome sequencing while being able to provide a global insight into the genetic material of organisms, is unable to always help in identifying the genetic causes of localized genetic disorders, such as rare Mendelian diseases of specific tissues (Wortmann et al. 2015; Turro et al. 2020).

Other recent works also reported on RNA-sequencing (RNA-Seq) gene expression (GE) data being a useful additional source of information for finding the genetic causes of such rare diseases (Cummings et al. 2017; Kremer et al. 2017; Bamshad et al. 2019; Mertes et al. 2021; Murdock et al. 2021; Yépez et al. 2021).

These works used RNA-Seq GE data in different ways but in our work, we focus only on finding outliers in RNA-Seq GE count data. To our knowledge, only Brechtmann et al. (2018), Salkovic et al. (2020), and Salkovic and Bensmail (2021) developed models for specifically tackling the problem of finding outlier counts in RNA-Seq GE data for the purpose of identifying the genetic causes of Mendelian disorders. Both used the negative binomial distribution (NBD) to model the data.

Other researchers relied on general methods for detecting outliers and methods developed for other purposes. For example, Cummings et al. (2017) calculated simple *z*-scores of log-transformed and normalized counts, while Kremer et al. (2017) also used a *z*-score-based approach in conjunction with a statistical significance test from DESeq2 (Love et al. 2014), which was originally designed for differential expression not outlier detection. However, Brechtmann et al. (2018) have shown that building a model specifically for outlier detection with incorporated confounder control can give better results: their model detected six out of six validated pathogenic events as outliers, while Kremer et al. (2017) detected only four.

Salkovic et al. (2020) and its improved version Salkovic and Bensmail (2021) focused on data with outliers, neglecting confounder control while suggesting that confounder control could be implemented downstream. However, they did not provide any concrete recommendations. All of these outlier-oriented models have downsides. When it comes to the OUTRIDER (Outlier in RNA-Seq Finder) model by Brechtmann et al. (2018), it can be deduced that its authors were forced to use an autoencoder (AE) to control for confounders, as they had been unable to obtain good results using a simpler dimensionality reduction method such as principal component analysis (PCA). They themselves suggest that PCA is not suitable for the NBD loss function that they used for training their tool. Furthermore, as the AE they use is a denoising AE, in order to determine the latent dimension of the AE they relied on artificial noise injection with somewhat arbitrary characteristics: a mean of log(3) and a standard deviation of log(1.6), with a $10^{-2}$ frequency of injection. Moreover, in order to prevent convergence issues while training they had to initialize their model parameters manually, and monitor that the parameter values are between certain numerical bounds. Finally, although the NBD loss function they constructed is mathematically sound, we can say that the process of deriving it is somewhat laborious because they did not include it in the paper’s main body. Instead, they detailed the numerous steps in the Supplementary material. Similar complexity issues affected the updating of the parameters of the AE’s decoding matrix which is tightly tied to the loss function. Another drawback of their overall approach is that they tested the performance of their method on datasets that were derived from real datasets by injecting artificial outliers using a simple z-score-based procedure without mimicking confounding effects. However, the main stated reason for developing OUTRIDER was for it to detect outliers masked by confounding effects, not simple outliers. Due to the complexity of their model, they did not inject artificial outliers masked by confounders.

Having said that, we have to acknowledge that their model performs well on the real datasets they tested it against, as well as datasets with artificially injected outliers: at the time of writing this work their model was state of the art. As Salkovic et al. (2020) and Salkovic and Bensmail (2021) used OUTRIDER in their performance comparisons, from their results we can say that OUTRIDER seems to perform best on data with underexpressed outliers.

As far as OutPyR by Salkovic et al. (2020) is concerned, the main downsides of their Bayesian model are its computational complexity leading to very long execution times and the lack of confounder control, which prevents the model from being performant on datasets with outliers that are masked by confounding effects. Although the authors suggest performing confounder control downstream, i.e. on the *P*-value matrix that their model produces, they fail to show a concrete way of how to perform it.

Still, while their model is based on the NBD distribution, due to its Bayesian nature it does not impose unique, rigid values on the parameters of the model, but instead the values are sampled from a loose posterior distribution of the parameters using a recently discovered method of inferring the dispersion parameter of the NBD through a particular form of Gibbs sampling (Zhou and Carin 2015). This allows their model to incorporate uncertainty in their reported *P*-values, which can be useful for downstream analysis. Also, they show that their model performs well in some settings with datasets containing a small number of samples and artificially injected outliers, as well as a synthetic NBD dataset with a large number of samples having no confounding. Salkovic and Bensmail (2021) with their Bayesian OutPyRX model further improved the OutPyR model by extending it with novel parameters which they utilize for developing a novel outlier score. Their model has multifold decrease in execution time compared to OutPyR, while also obtaining much better performance in their experiments involving real datasets with artificially injected outliers. However, they again use outliers with *z*-scores of only 3 and 4, neglecting 2. Although, similar to OutPyR, they do not perform any confounder control, nevertheless they get good results on datasets with a large number of samples, significantly outperforming OUTRIDER, both its AE version, as well as its PCA and PEER versions, all of which do perform confounder control.

### 1.1 Our contribution

In order to improve on results and the methodology of prior research efforts, in this article, we propose a novel method, OutSingle (Outlier detection using Singular Value Decomposition) for outlier detection in count data. The method can be decomposed into two steps that have separate but complementary roles.

The first step is simply log-transforming the data and then calculating gene-specific *z*-scores. The second step, which is our main contribution, is a confounder control method for *z*-score matrices (such as the one calculated in the first step) that relies on the recently discovered method for denoising matrices by finding an optimal hard threshold (OHT) for discarding singular values obtained through singular value decomposition (SVD) (Gavish and Donoho 2014).

In comparison to OUTRIDER, by using SVD and OHT we evade the need to implement an *ad hoc* AE, and thus avoid deriving complicated loss functions, training using artificial noise, and performing repeated inference in order to find the latent AE dimension. Both SVD and OHT are deterministic.

When we apply our model on the benchmark dataset by Kremer et al. (2017) which contains real, biologically aberrant counts masked by confounder effects, we obtain results better than the previous state-of-the-art model OUTRIDER (Brechtmann et al. 2018) in a fraction of its running time.

Moreover, relying on the fact that both steps of our procedure are “invertible”, we implement an inverse procedure in order to inject artificial outliers masked by confounding effects. Due to the complexity of the OUTRIDER model, such a procedure was not even considered by Brechtmann et al. (2018).

In the following section, we give more details about our methods, in Section 3, we briefly describe the datasets we use to test the performance of our method, in Section 4, we discuss the results of those tests, and we conclude with Section 5.

## 2 Materials and methods

An RNA-Seq GE count dataset is a *J *×* N* matrix of counts *k_ji_* whose columns correspond to different samples i∈1,…,N, while rows correspond to different genes j∈1,…,J with J≫N. When it comes to datasets that are used for identifying rare Mendelian disease disorders, samples would correspond to same-tissue samples from different patients having a rare disease specific to that tissue.

### 2.1 Log-normal *z*-scores

Our assumption is that the counts *k_ji_* follow a log-normal distribution. Such modeling of count data is well known (see Robinson et al. 2010; McCarthy et al. 2012; Wu et al. 2013; Law et al. 2014; Zhu et al. 2019). Based on that assumption, we calculate gene-specific *z*-scores for every count in the matrix. First, we control for sequencing depth using sample-specific DESeq size factors *s*_*i*_ (Anders and Huber 2010): Then, we log-transform the controlled counts $c_{ji}=k_{ji}/s_{i}$: where ${\bar{c}}_{j}$ are gene-specific mean values of *c_ji_*.


(1) $$s_{i}=\underset{i}{\text{median}}\frac{k_{ji}}{{\left(\prod_{t=1}^{N}k_{jt}\right)}^{\frac{1}{N}}}$$

(2) $$c_{ji}=\frac{k_{ji}}{s_{i}},$$

(3) $$l_{ji}={\;\text{log}\;}_{2}\left(\frac{c_{ji}+1}{{\bar{c}}_{j}+1}\right),$$

Now, we can model the *l_ji_* values as being normally distributed and having gene-specific means ${\bar{l}}_{j}$ and standard deviations *λ_j_* as the following: we assume that our data are expressed as a matrix $L=(l_{ji})$ where j=1,…,J (genes), i=1,…,N (samples), and $\Lambda =\text{diag}\{\lambda_{1},\ldots ,\lambda_{J}\}$. Therefore, we can express every row of the matrix $l_{j.}$ as a vector having a normal distribution, i.e.


(4) $$l_{j.}∼N({\bar{l}}_{j},\lambda_{j})$$

This assumption is motivated by the fact that even the original counts *k_ji_* in a single gene can be seen as being generated by a single gene-specific underlying process. For example, Salkovic et al. (2020) use gene-specific NBD modeling. Thus, it is reasonable to expect that the *l_ji_* values will have gene-specific normal distributions.

Finally, we can calculate a *J *×* N z*-score matrix $\tilde{Z}$ (the reason for the tilde notation will be made clear in the next section), whose members ${\tilde{z}}_{ji}$ correspond directly to the original counts *k_ji_*: where *μ_j_* and *τ_j_* are the gene-specific means and standard deviations of *l_ji_* values. By doing this, we have standardized the whole matrix. It is well known that many algorithms perform best on data that is standardized, and we use that fact in the next step, by applying SVD on the obtained *z*-score matrix.


(5) $${\tilde{z}}_{ji}=\frac{l_{ji}-\mu_{j}}{\tau_{j}},$$

### 2.2 OutSingle: confounder control using SVD and OHT

Here, we develop a confounding control procedure based on applying SVD on the matrix $\tilde{Z}$. The main benefit of using SVD for confounder control over an AE is the ease of implementation, as SVD is available in many programming languages and does not require extensive *ad hoc* setting up the way an AE would require. Another benefit is speed due to the SVD being an established method and therefore there are many optimizations in its implementations. We used SciPy’s (Virtanen et al. 2020) implementation. Perhaps, the most important benefit is that we do not have to perform repeated AE training in order to find the optimal latent AE dimension. Instead, we can rely on an almost instantaneous procedure which is described in Section 2.2.1.

We consider the matrix $\tilde{Z}$ as being a perturbation (hence the tilde notation) of a low-rank matrix **Z** (Stewart 1998): where **E** is a Gaussian noise matrix. The matrix **Z** is also called “signal” (Stewart 1998). In the following, we will show what the expressions of **Z** and **E** are. The *z*-scores corresponding to biological outliers will be contained in the noise matrix **E**. Because they have an extremely sparse impulse-like distribution, they cannot be considered part of the signal. The signal matrix **Z**, on the other hand, can be thought of as “capturing” the confounding effects because it has an underlying coherent structure that is distinct from both Gaussian noise and outliers. Using SVD, we can decompose $\tilde{Z}$ as follows: where $\tilde{U}$ and $\tilde{V}$ are orthogonal matrices with *J *×* J* and *N *×* N* dimensions and $\tilde{\Sigma }$ is a rectangular diagonal *J *×* N* matrix, having non-negative values on the diagonal called singular values (i.e. $\tilde{\Sigma }={\text{diag}}_{J\times N}({\tilde{\sigma }}_{1}^{2},\ldots ,{\tilde{\sigma }}_{N}^{2})$). Specifically, columns of $\tilde{U}$ and $\tilde{V}$ in the singular value decomposition of $\tilde{Z}$ always form an orthogonal set with no assumption on $\tilde{Z}$. Therefore they capture the projection space and all information is captured by $\tilde{\Sigma }$ including perturbation and noise, which means that there is a sensitivity in the singular value $\tilde{\Sigma }$. The singular values of $\tilde{\Sigma }$ are in descending order from the top left value to the bottom right value on the diagonal. That is the standard way of ordering singular values.


(6) $$\tilde{Z}=Z+E\;,$$

(7) $$\tilde{Z}=\tilde{U}\tilde{\Sigma }{\tilde{V}}^{T},$$

Considering this descending order of singular values, we can say that, theoretically, if we knew only the rank *r* of the low-rank signal matrix **Z**, we could split $\tilde{\Sigma }$ into two matrices that would directly correspond to **Z** and **E**: where Σ would then be an r-rank matrix, having zeroes in the lower right part, while $\Sigma_{E}$ would be a matrix of rank *N − r*, having zeroes in the top part. Using the previous set up, we have Approximating the original matrices **Z** and **E** is then trivial: which proves (6).


(8) $$\tilde{\Sigma }=\Sigma +\Sigma_{E},$$

$$\begin{array}{c} \Sigma =\left(\begin{array}{cc} \Sigma_{\left[r,r\right]}^{r} & 0_{\left[r,N-r\right]}\\ 0_{\left[J-r,r\right]} & 0_{\left[J-r,N-r\right]} \end{array}\right)\;\Sigma_{E}=\left(\begin{array}{cc} 0_{\left[r,r\right]} & 0_{\left[r,N-r\right]}\\ 0_{\left[J-r,r\right]} & \Sigma_{\left[J-r,N-r\right]}^{n} \end{array}\right)\\ \Sigma_{\left[r,r\right]}^{r}=\left(\begin{array}{cccc} \sigma_{1}^{2} & 0 & 0 & 0\\ 0 & \sigma_{2}^{2} & 0 & 0\\ \vdots & \vdots & \ddots & \vdots \\ 0 & 0 & \cdots & \sigma_{r}^{2} \end{array}\right)\;\Sigma_{\left[J-r,N-r\right]}^{n}=\left(\begin{array}{cccc} \epsilon_{1}^{2} & 0 & 0 & 0\\ 0 & \epsilon_{2}^{2} & 0 & 0\\ \vdots & \vdots & \ddots & \vdots \\ 0 & 0 & \cdots & \epsilon_{N-r}^{2}\\ 0 & 0 & \cdots & 0 \end{array}\right) \end{array},$$

$$\begin{array}{c} \tilde{Z}=\tilde{U}(\Sigma +\Sigma_{E}){\tilde{V}}^{t}\\ =\tilde{U}\Sigma {\tilde{V}}^{t}+\tilde{U}\Sigma_{E}{\tilde{V}}^{t}. \end{array}$$

(9) $$Z\approx \tilde{U}\Sigma {\tilde{V}}^{T}$$

(10) $$E\approx \tilde{U}\Sigma_{E}{\tilde{V}}^{T}$$

In the remainder of the work, we will treat these two approximations as equality in order to reduce the amount of symbols we use for notation. The rank *r* can be considered as a “cutoff”, because by keeping only the first *r* singular values and dismissing the rest, the noise is removed and only signal is kept.

#### 2.2.1 Optimal hard threshold

**Figure 1:**
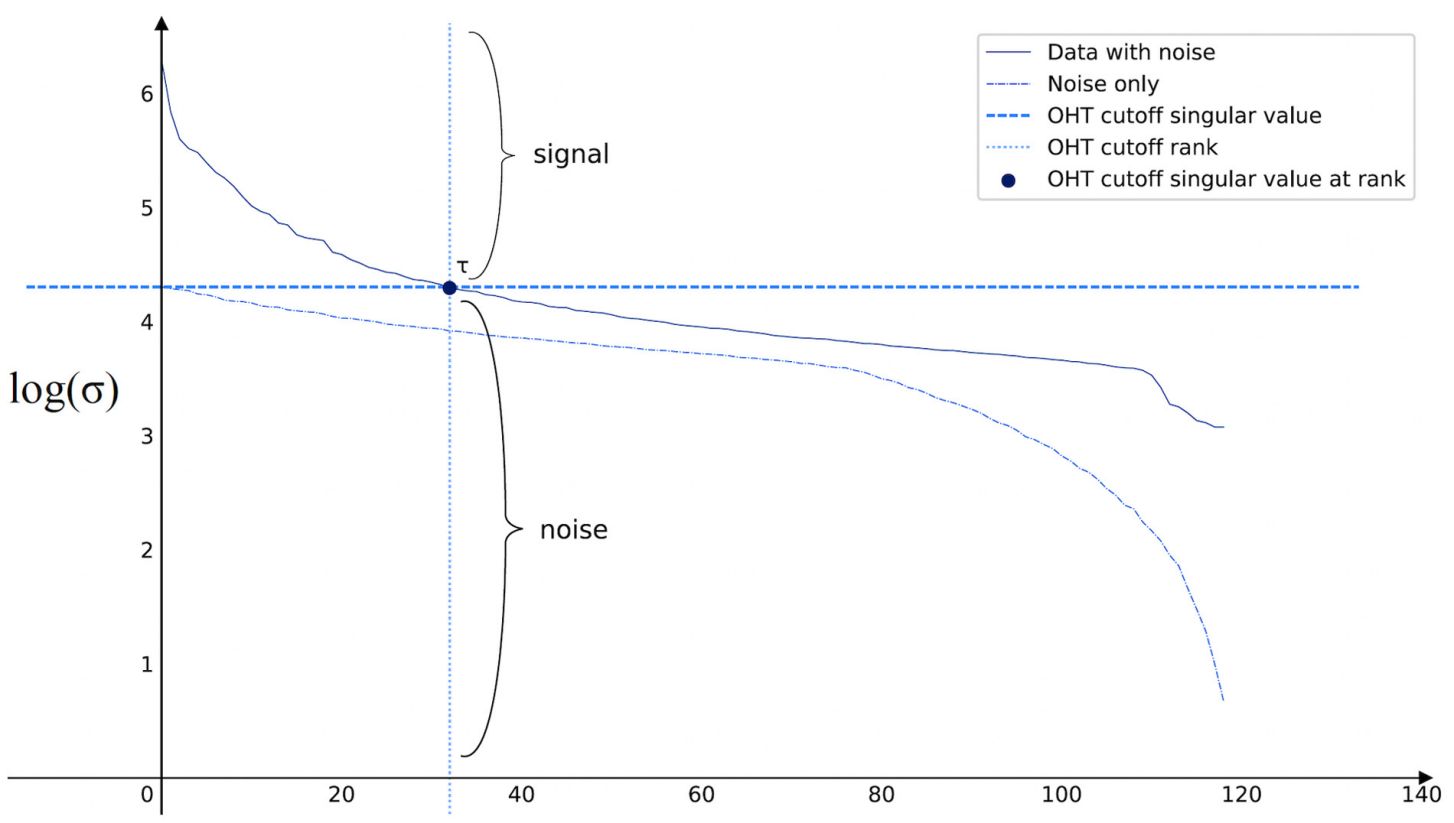
Schema of how cutoff value is determined to find the low-rank matrix

Gavish and Donoho (2014) have found an optimal method to remove the noise **E** from an SVD-decomposable matrix $\tilde{Z}$ by finding the rank *r* (i.e. singular value cutoff which they call OHT) of the low-rank matrix Σ (Fig. 1). The assumption under which their model is valid is that the **E** has the following property (Brunton and Kutz 2019): where *γ* is a scalar corresponding to the magnitude of the noise and $E_{\text{SND}}$ is a matrix of values that have a standard normal distribution (SND). We can assume that in our setting this is the case, as majority of real datasets are affected by normally distributed noise, including RNA-Seq GE datasets.


(11) $$E=\gamma E_{\text{SND}}\;,$$

They have proposed an exact solution for finding the rank *r* for the case where *γ* is not known as follows: where $\sigma_{\text{median}}$ is the median *σ* value of the values on the diagonal of $\tilde{\Sigma }$ and β=N/J. ω(β) is the following function: where: and $\mu_{\beta }$ is the solution to:


(12) $$r=\omega (\beta )\sigma_{\text{median}}\;,$$

(13) $$\omega (\beta )=\lambda (\beta )/\mu_{\beta }\;,$$

(14) $$\lambda (\beta )={\left(2(\beta +1)+\frac{8\beta }{(\beta +1)+{\left(\beta^{2}+14\beta +1\right)}^{1/2}}\right)}^{1/2}\;,$$

(15) $$\int_{{\left(1-\beta \right)}^{2}}^{\mu_{\beta }}\frac{{\left(\left({\left(1+\sqrt{\beta }\right)}^{2}-t\right)\left(t-{\left(1-\sqrt{\beta }\right)}^{2}\right)\right)}^{1/2}}{2\pi t}dt=1/2\;.$$

The benefit of this procedure, which is equivalent to finding the latent dimension of an AE, is that it does not require repeated calculations for all possible values of *r*, as, *r* might theoretically, range from 1 to min(J,N). It only requires solving Equations (12)–(15), for which the greatest “challenge” is the fact that the integral in (15) does not have a closed-form solution, but can be successfully and quickly approximated numerically using a Python tool, optht, (https://github.com/erichson/optht) for calculating the values above and we used it in our implementation.

#### 2.2.2 Final outlier score calculated by OutSingle

Similar to the derivation of the *z*-scores in Section 2.1, for OutSingle we assume that the members *e_ji_* of **E** follow gene-specific normal distributions and then proceed with deriving *z*-scores ${\hat{z}}_{ji}$ analoguous to (4) and (5). We omit the derivation steps for brevity. Our rationale for doing a similar derivation is the fact that both outliers masked by confounding effect and unmasked outliers, if any, will be contained in the **E** matrix, as, by definition, they cannot be a part of the signal matrix **Z**. However, the true outliers will still be outliers with respect to a single gene, as the noise levels in **E** for different genes could be different. Moreover, even a masked outlier is only considered an outlier due to its deviation with respect to a single gene, irrespectively from other genes.

Finally, in order to obtain a *P*-value score ${\hat{p}}_{ji}$ that is directly comparable with the *P*-value scores of OUTRIDER and OutPyR(X), we perform the following calculations for every element ${\hat{z}}_{ji}$: where Φ is the cumulative distribution function of SND. Because biological confounding effects cannot be completely eliminated, the expression levels of multiple genes in the same sample are associated. The Benjamini–Yekutieli false discovery rate (FDR) approach (Benjamini and Yekutieli 2001), which holds under positive dependency, was used to correct multiple tests.


(16) $${\hat{p}}_{ji}=2\cdot \min\{\Phi ({\hat{z}}_{ji}),1-\Phi ({\hat{z}}_{ji})\}\;,$$

### 2.3 Injecting artificial outliers by “inverting” the outlier detection procedure

An added benefit of using log-normal *z*-scores and SVD for outlier detection is that, due to their “invertibility”, they can be used for injecting artificial outliers that are masked by confounding effects. In terms of time complexity, the whole “inverted” procedure is similar to the original procedure.

We have already shown in (9) and (10) that by using OutSingle it is possible to obtain the low-rank matrix **Z** that contains confounding effects. We can add artificial outliers to it by generating an artificial noise matrix analogous to **E** in which we specify the exact locations of outliers as well as their magnitude. The resulting matrix will be equivalent to the $\tilde{Z}$ matrix from (6).

From such new $\tilde{Z}$ matrix, relying on (5), we can easily obtain new *l_ji_* values, and consequently, new *k_ji_* values. That way we obtain the final dataset containing all of the artificial outliers masked by confounders.

## 3 Datasets

For the purpose of evaluating performance, we ran our methods and competing methods on 18 datasets that we derived from two base datasets by injecting outliers using the procedure outlined in Section 2.3. The two base datasets are:

1. Kremer-119, a dataset of transcriptome gene expression counts from skin fibroblast tissues of 119 patients with rare diseases published by Kremer et al. (2017). The patients from that study were suspected of having some rare mitochondrial disorder. Nonexpressed genes were filtered out utilizing the pipeline of Brechtmann et al. (2018), resulting in retaining 10 556 out of 27 682 genes.
2. Using Brechtmann et al. (2018) approach for filtering genes, we removed out samples with a low RNA integrity number (< 5.7) from the GTEx dataset. Fragments per kilobase per millions of reads (FPKM) values were obtained with DESeq2 (Love et al. 2014) where the gene length was defined as the aggregated length of all the exons. They also filtered out expressed genes, by keeping genes for which at least 5% of the samples had a FPKM value greater than 1 and also discarded genes that had zero counts in more than 75% of the samples.
3. Murdock-125, a dataset of transcriptome gene expression counts from blood tissues of 125 patients with rare diseases recruited by Baylor College of Medicine clinical Undiagnosed Diseases Network site (UDN) published by Murdock et al. (2021a). The majority of patients were in the pediatric age group, and nearly half had a primary neurologic phenotype, consistent with the overall UDN historical proportions.

### 3.1 Derived datasets

Out of every base dataset we created derived datasets by injecting outliers following the procedure described in Section 2.3. Similar to the approach of Brechtmann et al. (2018), Salkovic et al. (2020), and Salkovic and Bensmail (2021), we created three separate groups of derived datasets: one that had only overexpressed outliers, another one that had only underexpressed outliers and a third group that had both underexpressed and overexpressed outliers in equal proportions using three schemes:

1. Overexpressed outliers: the derived dataset would contain only injected outliers whose value was greater than the mean/mode of a particular gene.
2. Underexpressed outliers: outlier value was less than the mean/mode of a particular gene.
3. Overexpressed and underexpressed outliers: outliers are generated using a ratio of 50%/50%.

In each of those three groups, we had three derived datasets that had different magnitudes of outliers: 6, 7, and 8. So, in total, we created nine derived datasets for every base dataset. We injected randomly a single outlier in every sample, due to Mendelian disorders being caused by a single gene. The outliers were injected as follows:

1. an outlier could be injected in any cell of the count matrix, if possible;
2. frequency of injection was $10^{-4}$.

The magnitudes of outliers were different than the ones chosen by Brechtmann et al. (2018) (2, 3, 4, and 6) and Salkovic et al. (2020), Salkovic and Bensmail (2021) (2, 3, and 4) because they used a simplistic approach without any consideration for confounding effects. In contrast, with our z-score-based approach, using the well-known order statistic for the SND, it is possible to calculate precisely the expected values of the lowest and highest z-score value in a matrix. Those extreme values are directly related to the number of elements in the matrix *J *×* N*, and for the datasets we used their magnitude was approximately 5, so we chose 6 as the lowest magnitude of the injected outliers. We did not use magnitudes >8 for brevity as they did not contribute significant added value.

### 3.2 Simulated datasets

To study the performance of OutSingle, we generated a 100-sample synthetic dataset using inferred *p_j_* and *r_j_* values from the filtered gene subset of the Kremer et al. (2017) full dataset (all 119 samples) together with a negative binomial random generator in order to get artificial data that are similar to Kremer et al. (2017) data (see Salkovic and Bensmail 2020). As there are no confounder effects in this dataset, even though the number of samples is high (100) OutSingle should be able to compete successfully with the other methods.

### 3.3 *P*-value score function

In order to compare the performance of OutSingle against other methods, we ran both OutSingle and other methods on the aforementioned derived datasets of all base datasets, estimated their model parameters and finally calculated the *P*-values for every count based on those parameters, using functions provided by the methods. Similar to OutSingle’s *P*-values, these *P*-values can be treated as an outlier score: the smaller the *P*-value, the greater an outlier a particular count is for a particular method. Ideally, the smallest *P*-values would correspond directly to actual injected outliers, however, in reality, none of the methods were detecting outliers perfectly. Therefore, to accurately compare the methods against each other, we did not use absolute *P*-values, but we instead ranked *P*-values of each method separately from the smallest to the largest. In that way, the *P*-value ranks were identical between different methods, and thus directly comparable. The method that would have actual injected outliers among the lowest *P*-value ranks would be the best-performing method.

We have used the FDR Benjamini and Yekutieli (2001) which is the expected proportion of erroneous rejections among all rejections. In Multiple testing, regulating the FDR regulates the conventional FEW for which proportion of mistakes is controlled instead when many of the tested hypotheses are rejected to or smaller than 0.05.

### 3.4 Alternative control methods

We compared OutSingle’s performance on the derived datasets against (i) the novel AE-based OUTRIDER model introduced by Brechtmann et al. (2018), denoted by OUTRIDER-AE; (ii) the alternative model that OUTRIDER supports using PEER (Stegle et al. 2012) for confounder control, denoted by OUTRIDER-PEER; and (iii) another alternative model that OUTRIDER supports which uses PCA for confounder control, denoted by OUTRIDER-PCA. For OUTRIDER-AE, autoencoder preprocessing was used to model covariations while PEER and PCA were used to produce loadings in the lower dimension instead of AutoEncoder to produce OUTRIDER-PCA and OUTRIDER-PEER. The design of OUTRIDER-AE, OUTRIDER-PEER and OUTRIDER-PCA was done as suggested by Brechtmann et al. (2018). For OUTRIDER-AE, AutoEncoder preprocessing was used to model covariations while PEER and PCA were used to produce loadings instead of AE which was used with OUTRIDER and produced OUTRIDER-PCA and OUTRIDER-PEER.

### 3.5 Implementation

The code for OutSingle is implemented in Python using widely available packages. It has been tested on Linux and Windows. Several options can be modified by supplying a settings file. OutSingle can take advantage of multiple cores, following a multi-process approach. It supports progress reporting, trace saving to disk, with recovery if the program is stopped during execution. It uses pre-defined, but easily modifiable, pseudorandom number generator (PRNG) seeds, allowing for experiment reproducibility. In the case of using multiple cores, every (sub)process has its own PRNG, which allows computationally correct sampling. In addition, the full PRNG states for every process are saved along with the trace, providing resilient recovery even in the multi-process setting. If multi-processing is enabled it splits the data according to the desired number of virtual cores/processes, albeit smartly, taking into consideration not only the number of genes but also the sum of counts in each gene. OutSingle is available at https://github.com/esalkovic/outsingle.

## 4 Results

In order to accurately assess and compare the performance of all methods, we tested them on datasets that were based on the aforementioned synthetic and real datasets but contained artificially injected outliers.

### 4.1 Performance comparison of OutSingle and other relevant models on simulated datasets

For all datasets derived from the Simulated-100 base dataset, all methods were performing with an AUC-PR value close to 1, i.e. almost perfectly. This confirms our assumption that, provided there are no confounders, OutSingle can match the performance of other methods. Here using Davis and Goadrich (2006), since we are performing models comparison, then it does not matter because to use ROC/AUC or PR/AUPRC. In fact if ROC curve of one classifier is above the ROC curve of another classifier, the same also holds true for the PR curve, and vice versa. In this case, one classifier is better than the other for all thresholds in both the ROC and the PR space, and it usually does not matter whether one uses the ROC curve/AUC or the PR curve/AUPRC for comparing two classifiers.

**Figure 2:**
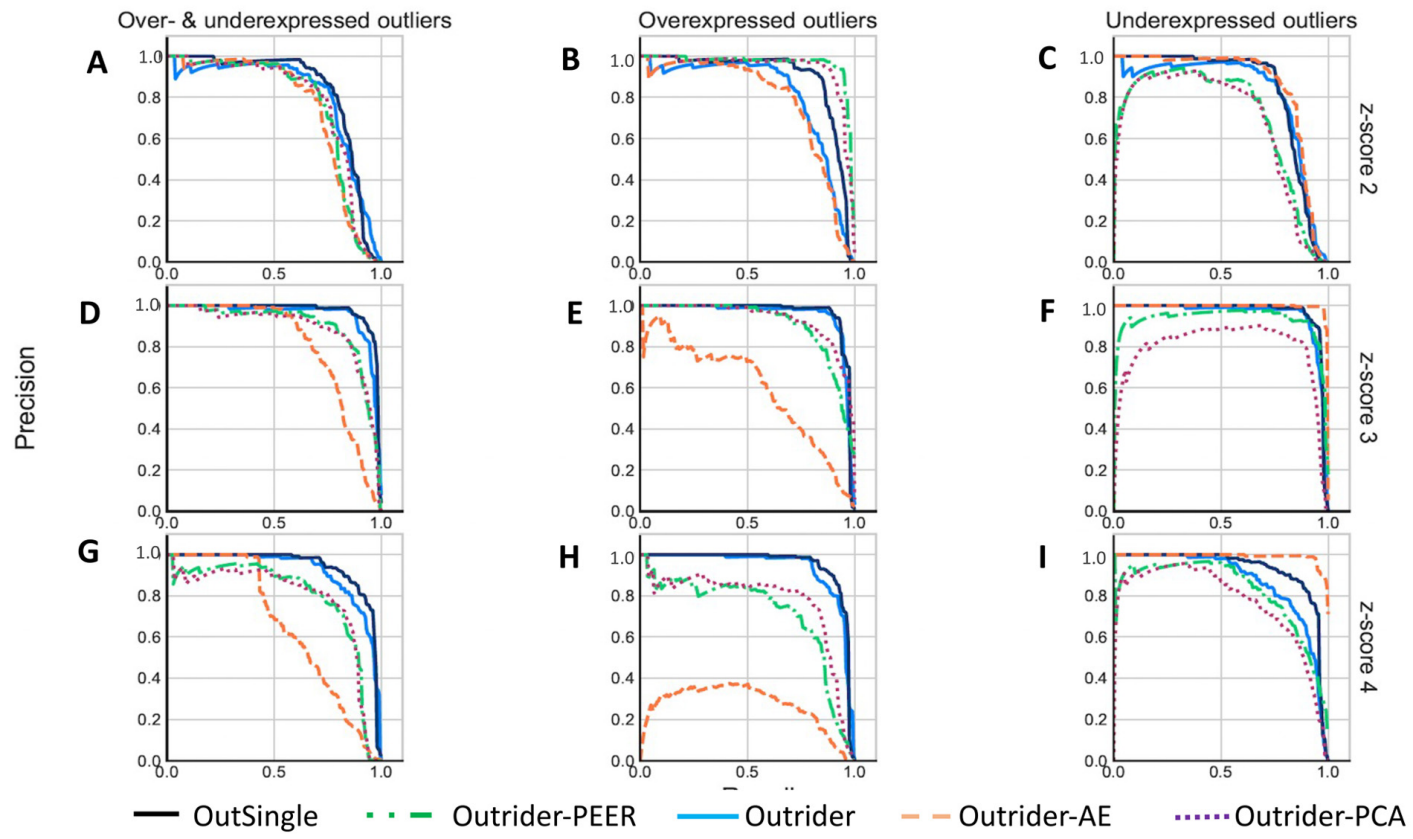
The proportion of simulated outliers among reported outliers (precision) plotted against the proportion of reported simulated outliers among all simulated outliers (recall) for the four approaches: Outsingle, Outrider-PEER, Outrider, Outrider-AE and Outrider-PCA

Figure 2 shows the proportion of simulated outliers among reported outliers (precision) plotted against the proportion of reported simulated outliers among all simulated outliers (recall) for increasing *P*-values up to FDR < 0.05 or decreasing absolute *z*-scores (PCA and PEER). Plots are provided for three simulated amplitudes (by row with simulated absolute *z*-scores of 2, 3, and 4 from top to bottom) and for three simulation scenarios (by column from left to right: aberrantly high and low counts, aberrantly high counts, and aberrantly low counts). The ranking of outliers was bootstrapped to yield 95% confidence bands.

### 4.2 Performance comparison of OutSingle and other relevant models on Kremer-115 and Murdock-125 datasets

**Figure 3:**
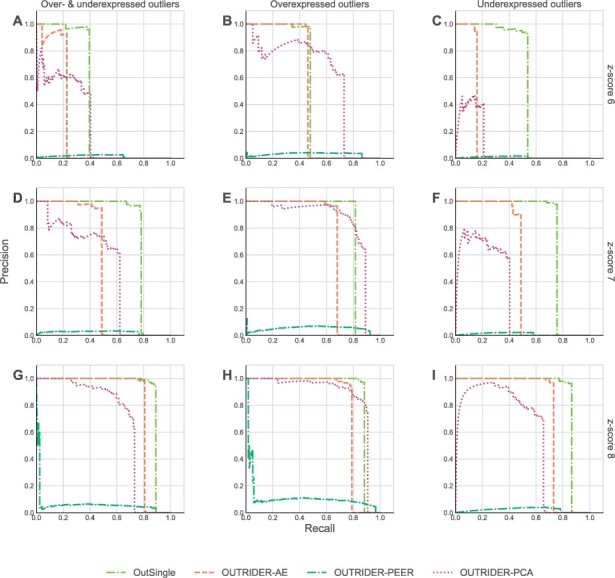
Precision versus recall plots of different methods (with different colors) for datasets derived from the base dataset Kremer-119

**Figure 4:**
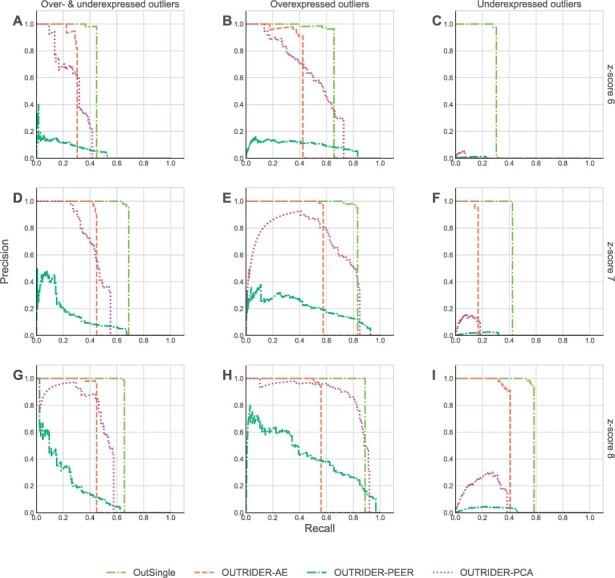
Precision versus recall plots of different methods (with different colors) for datasets derived from the base dataset Murdock-125

To compare different models on our artificially derived datasets, we use the same performance metric as in Brechtmann et al. (2018), Salkovic et al. (2020), and Salkovic and Bensmail (2021), namely the precision-recall (PR) curve and area under (PR) curve (AUC) value (Davis and Goadrich 2006). In our setting, precision refers to the ratio of the number of true outliers and the number of outliers as reported by a tool; whereas recall refers to the ratio of the number of true outliers among the reported outliers versus the number of all true outliers. In a single row, we plotted graphs for derived datasets with an identical *z*-score. In a single column we plotted graphs for derived datasets with an identical type of injected outliers: “Over- and Underexpressed” stands for “50% overexpressed outliers and 50% underexpressed outliers”, “Overexpressed” refers to outliers that were 100% overexpressed, and “Underexpressed” refers to outliers that were 100% underexpressed. Figures 3 and 4 show how different models performed on all derived datasets (Kremer-115 and Murdock-125) in terms of PR curves. The greater the area under a PR curve, the better its corresponding model is performing. As sometimes PR curves might not give a clear visual clue for which method is performing best.

**Figure 5:**
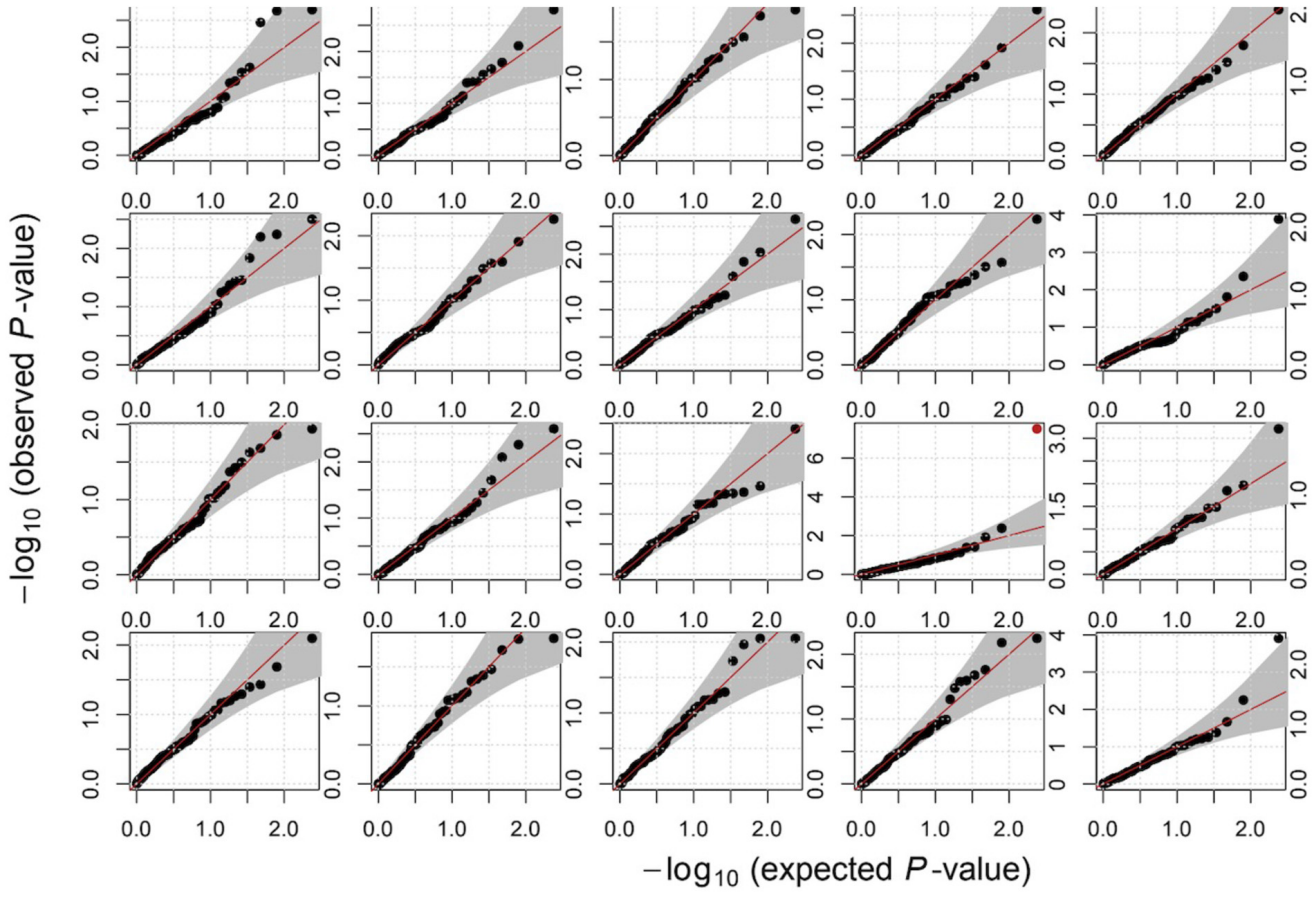
Quantile–quantile plot for 20 randomly selected genes from Kremer dataset

We then applied OutSingle on experimental data. Quantile–quantile plots for individual genes indicated that OutSingle reasonably modeled the count distribution (Fig. 5) on the Kremer datasets and that the resulting *P*-values can be used for detecting outliers.

In order to get a precise comparison among different methods we also calculated area under curve (AUC) numerical values for the PR curves of each method and each derived dataset, and grouped such values of all derived datasets of a particular base dataset in a single table. One such table is Tables 1 and 2, showing AUC-PR values of all methods for all derived nine datasets from the Kremer-119 base dataset and nine datasets from the Murdock-125 base dataset.

**Table 1:** AUC values for the various PR curves from Fig. 3.

	Over- and Underexpressed	Overexpressed	Underexpressed
z-score 6			
OutSingle	0.3900	0.4764	0.5275
OR-AE	0.2126	0.4620	0.1589
OR-PEER	0.0133	0.0294	0.0066
OR-PCA	0.2424	0.5933	0.0793
z-score 7			
OutSingle	0.7781	0.8152	0.7554
OR-AE	0.4818	0.6778	0.4813
OR-PEER	0.0226	0.0508	0.0102
OR-PCA	0.4894	0.8332	0.2696
z-score 8			
OutSingle	0.8882	0.8813	0.8630
OR-AE	0.8060	0.7863	0.7298
OR-PEER	0.0611	0.1156	0.0213
OR-PCA	0.6775	0.8677	0.5650

**Table 2:** AUC values for the various PR curves from Fig. 4.

	Over- and Underexpressed	Overexpressed	Underexpressed
z-score 6			
OutSingle	0.4465	0.6506	0.3035
OR-AE	0.2979	0.4137	0.0001
OR-PEER	0.0541	0.0880	0.0021
OR-PCA	0.2876	0.5236	0.0028
**z-score 7**			
OutSingle	0.6867	0.8297	0.4240
OR-AE	0.4466	0.5755	0.1669
OR-PEER	0.1174	0.1993	0.0060
OR-PCA	0.4503	0.6356	0.0221
z-score 8			
OutSingle	0.6559	0.8881	0.5823
OR-AE	0.4464	0.5582	0.4027
OR-PEER	0.1727	0.4156	0.0153
OR-PCA	0.4920	0.8416	0.0815

**Figure 6:**
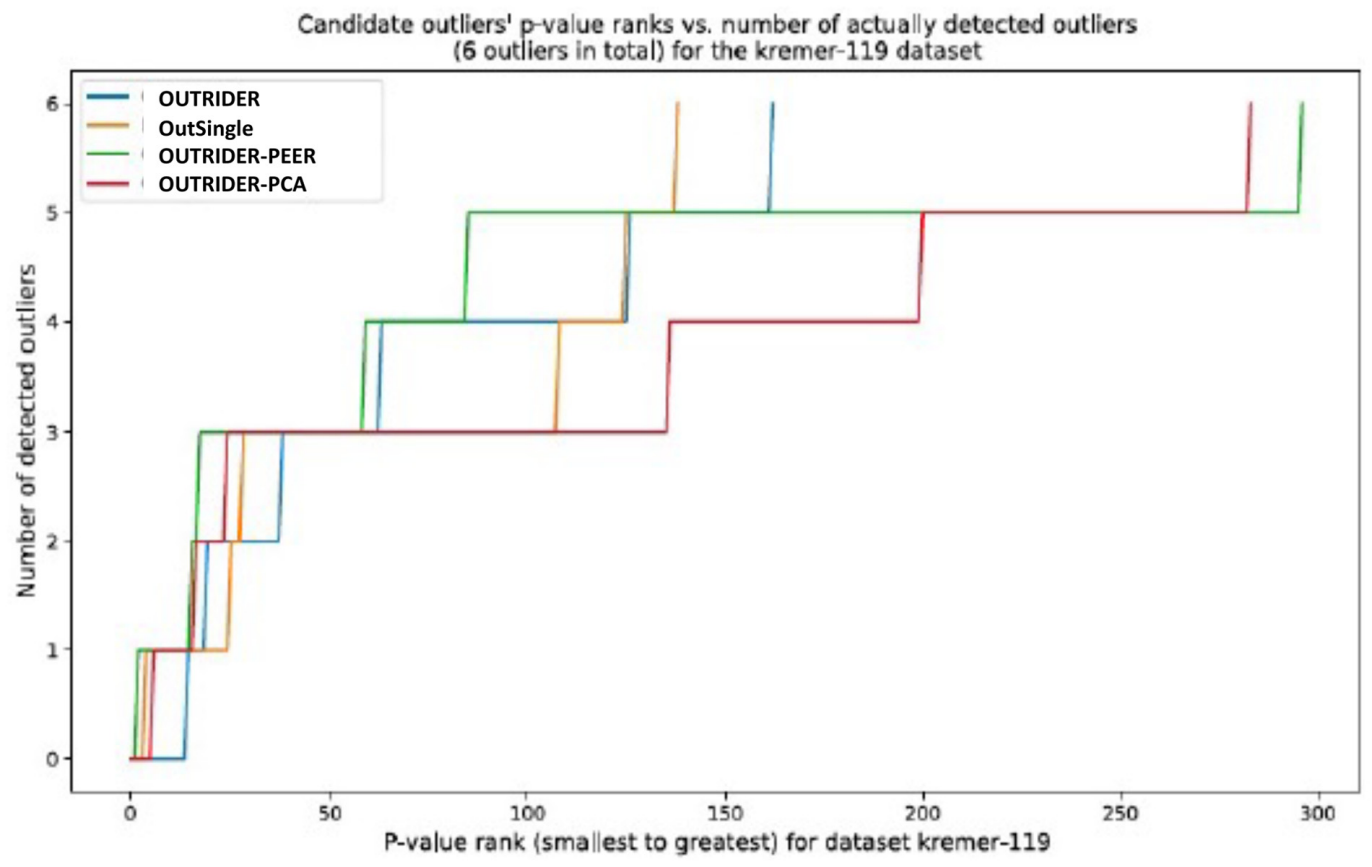
Number of aberrant events per sample by gene, colored by method

**Figure 7:**
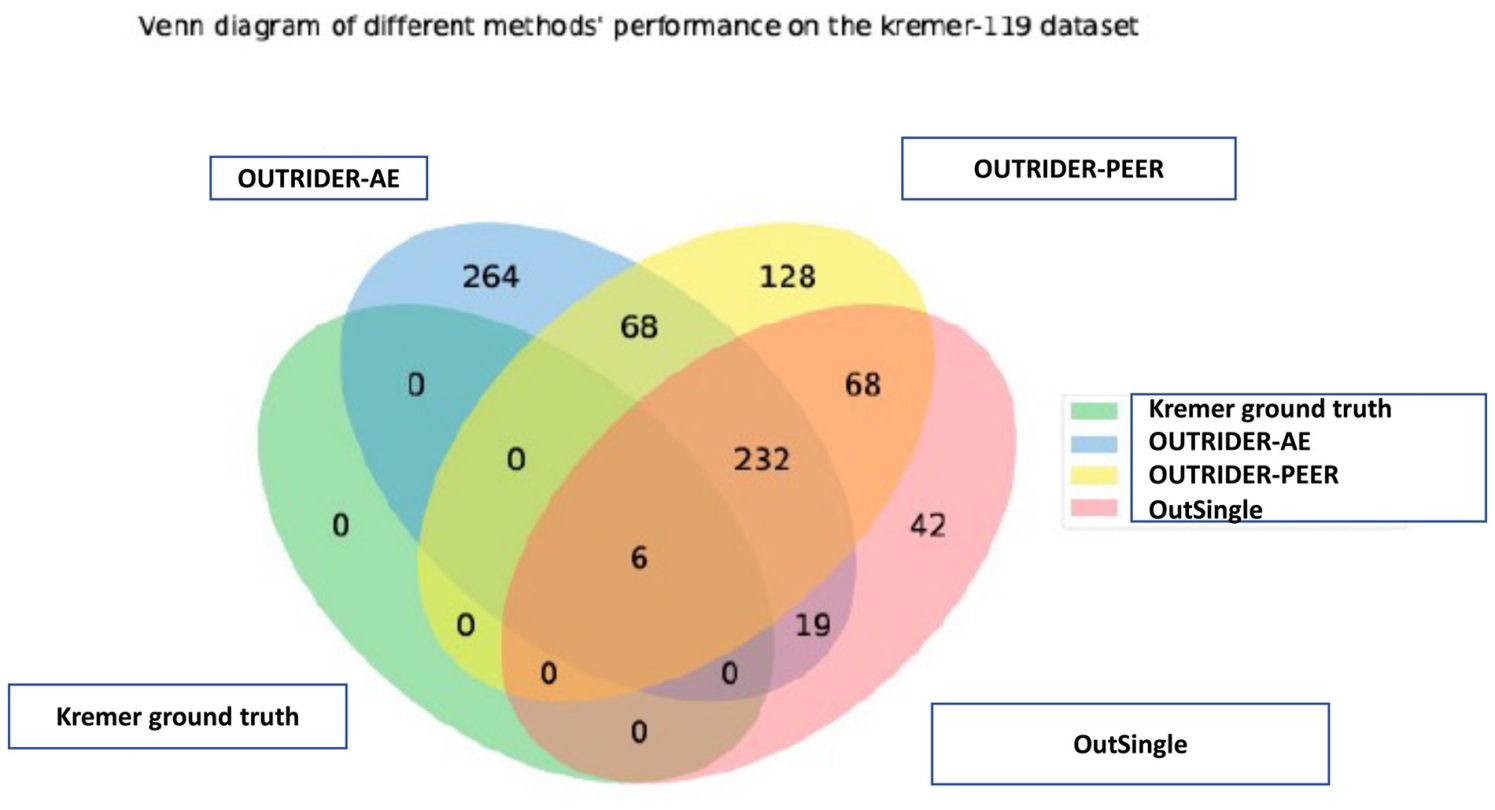
All the methods were able to recall all six pathogenic events validated by Kremer et al. On the Kremer dataset, OUTRIDER-PEER called 3.11 times more outliers than OutSingle, and OUTRIDER-AE called 3.38 more outliers than OutSingle. OUTRIDER-PCA did bad overall

In terms of the model having the lowest rank for the 6th biological outlier, it is undisputedly obtained by OutSingle: 122nd, followed by OUTRIDER-AE: 161st i.e. it performs more than 30% better than the previous state-of-the-art model. While the performance of OUTRIDER-AE, OUTRIDER-PEER, and OUTRIDER-PCA is good in detecting the first five outliers, but the last two approaches detect the 6th biological outlier, i.e. the last one, as the 270th and 280th (rank) candidate outlier, which is almost double the rank of OutSingle (122nd) (see Fig. 6). Applying OutSingle to the Kremer dataset resulted in a recall of 61 outliers (9.9%) identified by Kremer *et al.* on the basis of the 48 previously undiagnosed samples. Additionally, OutSingle detected 87 new expression outliers, of which 50 were downregulated. All the methods were able to recall all six pathogenic events (three expression outliers, one mono-allelic expression, and two splicing defects) validated by Kremer *et al.* On the Kremer dataset, OUTRIDER-PEER called 3.11 times more outliers than OutSingle, and OUTRIDER-AE called 3.38 more outliers than OutSingle. OUTRIDER-PCA did bad overall (Fig. 7). These results are consistent with the results from the simulations.

## 5 Conclusion

In this work, we have presented an algorithm we called “OutSingle” which is a contribution to the problem of detecting outliers in RNA-Seq GE dataset in order to help clinicians detect aberrant genes causing rare diseases in patients. Our contribution is an alternative solution to the Bayesian approach that performed well but was slow. Here, we introduced a novel fastway of normalizing RNA-Seq GE data which allowed us to use a much simpler ND approach instead of NB to model the data; and culminating with developing several models for confounder control, with which we have shown that with a rigorous and interpretable statistical approach in modeling the data in the first place, we could then use a straightforward and fast SVD based model for confounder control, improving on previous ad hoc, less interpretable models. Although the end goal of our research was detecting aberrant genes in a rare genetic disorder setting, it is reasonable to believe that our advancements can be utilized in other settings involving RNA-Seq data, such as differential expression analysis. We have shown that our model is able to detect outliers in small number of samples successfully, and even control for confounders, if not fully, at least to a significant extent. Moreover, our approach could be useful for single-cell RNA data as there have already been attempts to model this type of data using a model similar to the NB inference approach such as the recent work of Dadaneh et al. (2020).

## Notes

Financial Support: None declared.
